# Intern year in a developing country amidst COVID-19

**DOI:** 10.1080/10872981.2020.1785115

**Published:** 2020-06-26

**Authors:** Apurva Shrigiriwar, Tushar Garg

**Affiliations:** Department of Radiology, Seth GS Medical College & KEM Hospital, Mumbai, India

**Keywords:** COVID-19, intern, developing country, pandemic, healthcare

## Abstract

It was the middle of March when we were two weeks into our clinical rotation and just started to get the hang of the new hospital. When one morning after reaching the hospital and finishing our patient rounds and notes, we received a frantic message from our co-rotator, mentioning all clinical rotations for MD students have been suspended due to COVID-19, according to the new AMA guidelines. We immediately booked our flight tickets and flew back to Mumbai.

It was the middle of March when we were two weeks into our clinical rotation and just started to get the hang of the new hospital. When one morning after reaching the hospital and finishing our patient rounds and notes, we received a frantic message from our co-rotator, mentioning all clinical rotations for MD students have been suspended due to COVID-19, according to the new AMA guidelines. We immediately booked our flight tickets and flew back to Mumbai.

When we reached the airport, we were welcomed by familiar faces of our batchmates who had turned from nervous fourth-year students to confident interns (last year of medical school in India).

A day before joining, we got an email from our professor saying that we will be deployed to work on the hospital’s front lines to help manage the patients. A mixture of feelings were going through our brain, on the one hand ‘In the name of suffering humanity; with humility, compassion and dedication to the welfare of the sick according to the best of my ability and judgement’ we pledged to keep this oath, and on the other hand, we were worried about going back home to our parents with multiple comorbidities and putting their lives at risk.

In the last two months during this pandemic, we have worked at multiple hospital sites, government quarantine facilities, airports, and health camps. We have screened thousands of people entering the country at the airport, cared of hundreds of patients staying at quarantine centers, evaluated scared patients coming to cough, cold, fever outpatient departments (OPD) to get themselves checked, conducted door to door screening in the slums of Mumbai where more people live than the whole state of California within a 30-mile radius. All this while working in the scorching heat of Mumbai wearing full personal protective equipment (PPE) without any air-conditioning and feeling like a moving autoclave. During this time, we also collaborated with the local government bodies and traced contacts of positive patients and became the calming voice behind the disaster management helpline number- talking to people calling for help 24 × 7.

At one point, when there was a severe shortage of PPE kits, a group of interns sat down together after work (obviously with proper social distancing) with OHP sheets and made face shields which were then distributed to everyone working on the frontlines.

One day, a friend of ours noticed that there was a shortage of food for the children and indigenous families around the hospital, so we organized a food donation camp where we donated extra food daily from our food packets to those in need.

Even though this journey has been physically (rounding on patients at 5–6 different quarantine centers), mentally (working 24-hour shifts due to shortage of healthcare workers) and emotionally (coming face to face with death and broken families) draining, this journey has been worth it as we have been able to help thousands of patients who would have otherwise not received the quality of healthcare they deserve.

Although we are optimists, we do realize that at present, our healthcare system has started to show the cracks which were long hidden, there is a shortage of PPE, delay of salary payments to the same interns and residents who are working during this time and putting their life at risk, overflow of patients in the ICU leading to a shortage of beds and lack of testing kits to help access the severity of the pandemic in this country. Many of our residents, senior faculty, and staff member have tested positive for COVID-19, and some of them have not been able to find beds for themselves to get admitted to the hospital.

Are we ready to tackle this pandemic is our question? Will we ever be prepared to handle it? Will this become the new normal? There is no answer to this, but the smile on the faces of recovered patients and their family members is the best incentive that keeps us going!

